# Feasibility of photon-counting computed tomography as a novel imaging modality for challenging endodontic diagnostic tasks

**DOI:** 10.1038/s41598-023-33322-9

**Published:** 2023-04-17

**Authors:** Rocharles Cavalcante Fontenele, Fernando Fortes Picoli, Jader Camilo Pinto, Walter Coudyzer, Karla de Faria Vasconcelos, Amanda Farias Gomes, Joke Binst, Reinhilde Jacobs

**Affiliations:** 1 OMFS IMPATH Research Group, Department of Imaging and Pathology, Faculty of Medicine, University of Leuven, KU Leuven, Kapucijnenvoer 7, 3000 Leuven, Belgium; 2 Department of Oral & Maxillofacial Surgery, University Hospitals Leuven, KU Leuven, Leuven, Belgium; 3 Department of Oral Diagnosis, Division of Oral Radiology, Piracicaba Dental School, University of Campinas, Piracicaba, São Paulo Brazil; 4 Department of Dentistry, School of Dentistry, Federal University of Goiás, Goiânia, GO Brazil; 5 Department of Restorative Dentistry, School of Dentistry, São Paulo State University, Araraquara, São Paulo Brazil; 6 Department of Radiology, UZ Leuven, Leuven, Belgium; 7 Department of Dental Medicine, Karolinska Institutet, Stockholm, Sweden

**Keywords:** Dentistry, Diagnosis

## Abstract

Photon-counting computed tomography (PCCT) is an innovative technological advancement in relation to x-ray detectors which offers ultra-high-resolution images. The current study aimed to evaluate the visualization ability of PCCT compared to cone-beam computed tomographic (CBCT) devices for challenging endodontic diagnostic tasks. A reference image of an anthropomorphic phantom was acquired using an industrial micro-CT device. Thereafter, the phantom was scanned with three imaging devices, which included PCCT scanner (NAEOTOM Alpha) and two CBCT devices (3D Accuitomo 170 and NewTom VGi evo) having standard and high-resolution acquisition protocols. The diagnostic tasks involved visualizing fine endodontic structures (apical delta, narrow canal, and isthmus) and root cracks. Three experienced examiners assessed the images and were blinded to the PCCT and CBCT devices. Each image was rated according to a three-grade scale (appropriate, acceptable, or inappropriate) for the diagnostic tasks. In relation to fine endodontic structures grouped together, PCCT showed similar diagnostic performance compared to the reference image (*p* > 0.05). As for the CBCT devices, an excellent performance was only observed with the 3D Accuitomo 170 device at a high-resolution acquisition mode (*p* > 0.05). The visualization of root cracks was also better with 3D Accuitomo 170 compared to other devices (*p* < 0.05). Overall, PCCT and 3D Accuitomo 170 at a high-resolution setting showed similar performance for visualizing fine endodontic structures. In addition, the high-resolution CBCT protocol was superior for visualizing root cracks compared to both PCCT and other standard- and high-resolution CBCT protocols.

## Introduction

The assessment of tooth-related fine structures is a challenging diagnostic task which might impact the decision-making process related to endodontic treatment planning and clinical outcomes^1–3^. Three-dimensional (3D) cone-beam computed tomographic (CBCT) imaging has been regarded as a vital tool for performing such diagnostic tasks^1,2^. However, the diagnostic ability of CBCT devices is hampered due to the presence of certain limitations, such as low signal-to-noise ratio (SNR), low contrast resolution, and presence of artefacts generated from high-density materials (e.g. gutta-percha, sealers, and metal post)^4,5^.

Photon-counting CT (PCCT) has emerged as a novel imaging modality with a spatial resolution of up to 0.20 mm which is close to that of high-resolution (HR) CBCT devices (ranging from 0.08 to 0.125 mm)^6–10^. Unlike CBCT devices, it has an increased contrast resolution, high SNR, and high scanning speed. The major technological advancement of PCCT has been related to x-ray detectors, where sensors composed of a single layer of semiconductor diode have replaced the conventional x-ray detectors with scintillator material. This allows each absorbed x-ray photon to generate a charge cloud which is individually transported to the detector pixels by applying a bias voltage. Thereby, generating an electrical signal and dismissing an initial transformation into visible light which happens with CT detectors composed of conventional energy-integrating scintillator materials^9,10^. As no scintillator layer and reflecting lamellae exist in the PCCT detectors, their pixel size is smaller compared to conventional CT detectors which increases the image resolution^8,10^.

A recent in vitro study showed optimal performance of PCCT compared to CBCT imaging for different diagnostic tasks in the field of dentistry, which included detection of apical osteolysis, measurement of bone thickness and visualization of various dental structures and surrounding bone^9^. However, to our knowledge, the performance of PCCT for assessing challenging diagnostic tasks in endodontics has not yet been investigated. Hence, the aim of the present study was to evaluate the visualization ability of a PCCT device compared to two cone-beam computed tomographic (CBCT) devices for visualizing fine endodontic structures (apical delta, narrow canal, and isthmus) and root cracks, while using an industrial micro-CT device as a radiological reference. The null hypothesis adopted was that no difference exists between PCCT and CBCT devices for visualizing fine endodontic structures and root cracks.

## Materials and methods

This study was approved by the Local Ethics Committee, UZ/KU Leuven, Belgium (protocol number: NH019 2019-09-03) and conducted in compliance with the World Medical Association Declaration of Helsinki on medical research.

### Phantom

**Figure 1:**
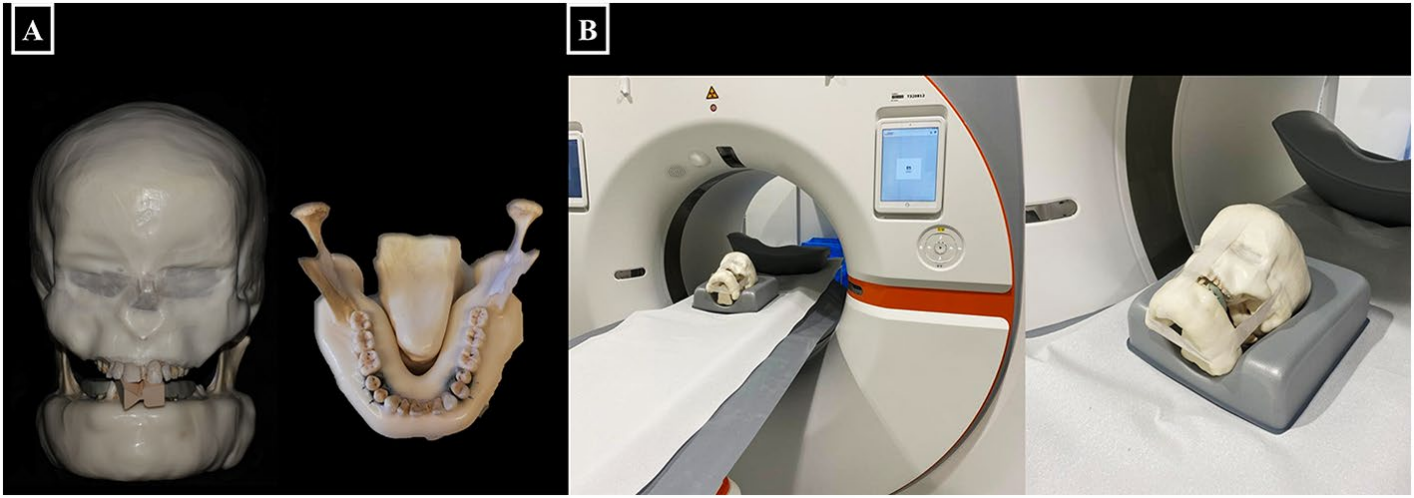
Anthropomorphic phantom scanning. (**A**) Anthropomorphic phantom composed of a dry human skull and a full-dentate mandible covered with Mix-D with a tongue model made with Mix-D; (**B**) Positioning of the anthropomorphic phantom in the PCCT NAEOTOM Alpha device.

An anthropomorphic phantom composed of a dry human skull and a full-dentate mandible was recruited for the image acquisition. The complete phantom was covered with a soft tissue equivalent (Mix-D) for simulating x-ray attenuation equal to that of human soft tissue, as described by Oenning et al^11^. In addition, a tongue model was also created with mix-D and placed at its corresponding position inside the phantom (Fig. 1A).

### Reference image acquisition

The dry human mandible was scanned with an industrial micro-CT device (Nikon XT H 225; Nikon Metrology Inc, Brighton, MI, USA) which acted as a radiological reference. The acquisition parameters were set at 200 kilovoltage-peak, 350 µA, 8 × 8 cm field of view (FOV) size, 2.5 mm Cu filtration, and 40 µm voxel size. An oral and maxillofacial radiologist having more than five years of experience confirmed the presence of endodontic fine structures (apical delta, narrow canal, and isthmus) and root cracks in the image. In case of uncertainty, a second senior dentomaxillofacial radiologist (R.J.) with over 30 years of experience was consulted. Both examiners confirmed the presence of apical delta, isthmus, and root cracks in the right mandibular 1^st^ molar and a narrow canal in the right mandibular 1^st^ premolar.

### Testing image acquisitions

The anthropomorphic phantom was scanned with two different CBCT devices (3D Accuitomo 170, J Morita, Kyoto, Japan and NewTom VGi evo, Cefla Dental Group, Imola, Italy) using standard (SR) and HR acquisition protocols, Afterwards, it was scanned with a PCCT device (NAEOTOM Alpha, Siemens Healthineers, Erlangen, Germany) (Fig. 1B). Table 1 summarizes the scanning parameters and effective dose to adults with each imaging device based on applied protocols. The estimated adult effective dose of the SR- and HR-CBCT protocols ranged from 0.03 to 0.13 mSv and 0.01 to 0.04 mSv, respectively. On the other hand, a higher estimated effective dose was noted with the PCCT device (0.85 mSv) based on the acquisition protocol for head scanning recommended by the manufacturers.

**Table 1:** Scanning parameters and estimated effective dose to adults of the different imaging devices.

Imaging devices	Protocol	Voxel (mm)	FOV (cm)	Tube voltage (kVp)	Exposure time * tube current (mAs)	Effective dose adults (mSv)
3D Accuitomo 170	SR	0.125	8 × 8	90	154	0.13
3D Accuitomo 170	HR	0.08	4 × 4	90	154	0.04
NewTom VGi evo	SR	0.125	8 × 8	110	20.96	0.03
NewTom VGi evo	HR	0.10	5 × 5	110	24.96	0.01
Photon-Counting CT	NA	0.20	8 × 8	120	64	0.85

SR, standard resolution; HR, high-resolution; NA, not applicable.

Dose conversion factor for adults CT head scan was 0.0029 mSv/mGy.cm (DOSE TQM, Qaelum, based on IRCP 103).

Dose conversion factor for adults for 3D Accuitomo 170 and NewTom VGi evo scans were 0.0076 and 0.008 mSv/dGy cm^2^, respectively.

The CBCT scans were reconstructed using a sharp kernel with an isotropic voxel size according to each acquisition protocol tested, as shown in Table 1. Regarding the PCCT, the scan reconstruction was done with the conventional filtered backprojection algorithm using sharp kernel (Qr76f. with bone window) onto slice thickness of 0.20 mm and a slice increment of 0.15 mm.

### Image registration

The images captured with both PCCT and CBCT devices were registered onto the reference micro-CT image using Amira software (Version 2019, Thermo Fisher Scientific, Waltham, MA, USA). This allowed to assess the endodontic structures and root cracks acquired from different devices at the same slice and anatomical level. The registration was performed with the application of a rigid voxel-based registration algorithm with mutual information, which functions by identifying maximum mutual information redundancy between image intensities of corresponding voxels found in different image datasets^12^.

### Image analysis

Five representative image reconstructions (at different anatomic levels) of the right mandibular first molar of the phantom, showing the apical delta, isthmus, and root cracks were selected for each imaging dataset using ImageJ software (U.S. National Institutes of Health, Bethesda, MD, USA). Also, five representative image reconstructions of the right mandibular first premolar showing the narrow canal were selected following the same methodology previously described, totaling up to 100 image reconstructions (5 reconstructions × 4 diagnostic tasks × 5 imaging datasets). Later, the image reconstructions were imported to Microsoft Office PowerPoint software (Microsoft Co., Redmond, WA, USA) and presented on a black background without image compression. The presentation consisted of 20 sets of images (5 reconstructions × 4 diagnostic tasks) showing the same image reconstruction of each diagnostic task acquired using different tested imaging modalities. The reconstructions were randomly displayed for each set of images, and the examiners were blinded to the imaging device.

Three oral and maxillofacial radiologists with more than five years of experience were recruited for performing the subjective image analysis. The examiners were calibrated and trained to identify different endodontic tasks on a set of unused images which were not a part of the study. Prior to image assessment, an experienced radiologist (R.C.F.) adjusted the brightness and contrast of all the images as inherent differences were observed in their histogram distribution. Although the examiners were blinded to the acquisition device and protocol, they were still aware of each diagnostic task and its location by indicating them onto the reference image which was provided next to the test images as a visual aid. The assessment was performed in a dimmed light environment using HR medical display MDRC-2221 (Barco, Kortrijk, Belgium).

The representative reconstruction from each set of images were rated on a three-point rating scale^13^ by comparing with the reference image, where (1) appropriate for visualization of the indicated structure; (2) acceptable for visualization of the indicated structure (structure distinguished by the examiner, but less clear compared to the reference image); (3) inappropriate for visualization of the indicated structure (structure indistinguishable by the examiner). All examiners assessed the data twice at an interval of 30 days for calculating intra- and inter-observer reliability.

### Statistical analysis

Data were analyzed using GraphPad Prism 5 (GraphPad Software Inc, La Jolla, CA, USA). Intra- and inter-examiner agreements were determined by the weighted kappa test and interpreted according to Landis and Koch’s classification^14^. The scoring was summarized based on the most frequent score (i.e., mode) provided by the examiners for each diagnostic task using different imaging modalities. Within each diagnostic task, Kruskal–Wallis and Dunn’s tests were conducted to compare the scoring of different devices. A *p* value of less than 0.05 was considered significant.

## Results

Table 2 shows in detail the results regarding the intra- and inter-examiner agreements. Summarizing, the intra- and inter-examiner agreements were almost perfect, ranging from 0.83 to 0.91 and 0.82 to 0.95, respectively.

**Table 2:** Intra- and inter-examiner agreements for the subjective image analysis.

Examiners	1	2	3
1	0.90	0.96	0.82
2		0.91	0.95
3			0.83

**Figure 2:**
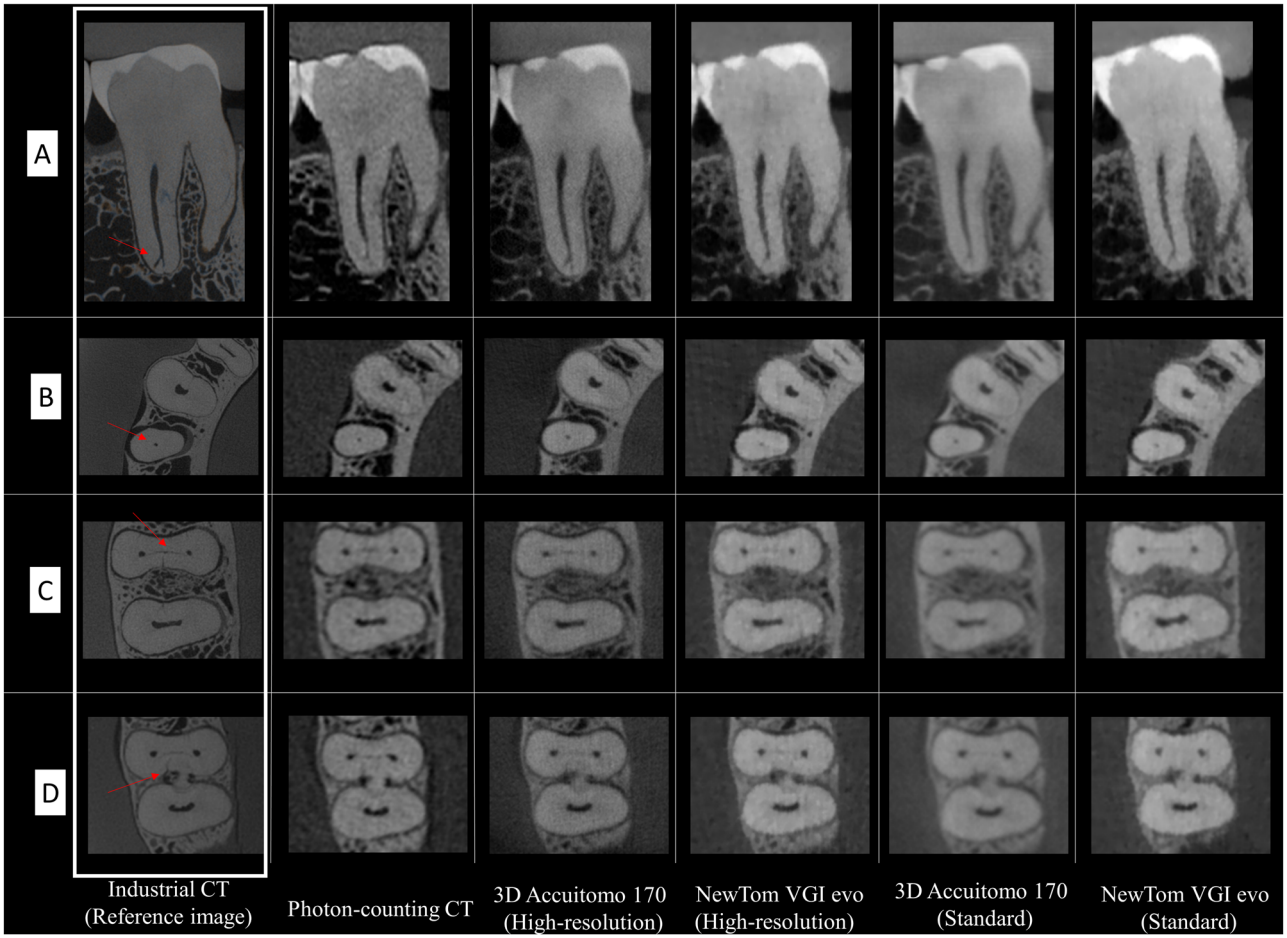
Representative imaging reconstructions of each diagnostic condition (as indicated by the arrows in the set of images of the Industrial CT—reference image) according to the different imaging modalities tested: (**A**) Apical delta; (**B**) Narrow canal; (**C**) Isthmus; (**D**) Root crack.

Table 3 describes the findings of the subjective image analysis based on mode values for visualizing fine endodontic structures and root cracks. Furthermore, Fig. 2 presents representative imaging reconstructions for the different tasks tested using the industrial micro-CT images as a reference. When the fine endodontic structures (delta, narrow canal, and isthmus) were grouped together, PCCT showed similar diagnostic performance compared to the reference image (*p* > 0.05). Amongst the two CBCT devices with variable protocols, HR 3D Accuitomo 170 was able to provide excellent diagnostic performance (*p* > 0.05) with appropriate visualization of the endodontic structures. Considering each endodontic structure individually, both PCCT and 3D Accuitomo 170 showed an appropriate score for visualizing narrow canal and isthmus (*p* > 0.05).

**Table 3:** Mode values for visualization assessment of fine endodontic structures (apical delta, narrow canal, and isthmus) and root cracks.

Diagnostic tasks	Reference image (Industrial CT)	Photon-counting CT	High Resolution		Standard Resolution	
			3D Accuitomo 170	NewTom VGi evo	3D Accuitomo 170	NewTom VGi evo
Apical delta	1	2–3	1*	3	2	2–3
Narrow canal	1	1*	1*	2	2	2
Isthmus	1	1*	1*	1—2*	2—3	3
Fine endodontic structures grouped^a^	1	1*	1*	2	2	2
Root crack	1	2	1*	3	3	3

*Indicates statistical similarity with the reference industrial CT device, which is shown as the reference image (1 – appropriate for visualizing the investigated diagnostic task, 2 – acceptable for visualizing the investigated diagnostic task, and 3 – inappropriate for visualizing the investigated diagnostic task).

^a^Indicates the fine endodontic structures (apical delta, narrow canal, and isthmus) grouped.

As for the root crack visualization, HR 3D Accuitomo 170 showed a better performance compared to PCCT and NewTom VGi evo regardless of its acquisition protocol *(p* < 0.05). In addition, root crack scoring was considered appropriate with HR 3D Accuitomo 170, whereas PCCT had acceptable scoring. On the other hand, SR 3D Accuitomo 170 and NewTom VGi evo with both SR and HR protocols showed an inappropriate visualization of the root cracks.

## Discussion

An accurate identification and visualization of fine anatomical and pathological structures is a prerequisite in the majority of diagnostic and treatment planning dental workflows. The following study is the first to experimentally evaluate the application of a PCCT device for visualizing hard-to-detect fine endodontic structures and root cracks. Although the proposed diagnostic tasks were challenging, the findings suggested that the PCCT device could act as a promising tool for diagnostic applications. The performance of PCCT for visualizing fine anatomical structures was equal to that of a micro-CT for most of the diagnostic tasks except for root cracks. Amongst the CBCT devices with variable parameters, only HR 3D Accuitomo 170 was able to provide a good diagnostic performance. The null hypothesis was rejected, considering that the PCCT was able to either appropriately or acceptably detect all challenging diagnostic tasks.

The performance of HR 3D Accuitomo 170 was better than PCCT for visualizing root cracks, which could be attributed to the fact that HR CBCT device offers better image clarity with a smaller voxel size (0.08 mm) compared to that of PCCT (0.2 mm). Furthermore, it is important to highlight that the images acquired with PCCT had a larger voxel size compared to crack sizes of the teeth being assessed (< 200 µm), which made it difficult to visualize the cracks. Although PCCT offers a higher effective radiation dose with an increased susceptibility to biological effects from ionizing radiation exposure compared to CBCT scans, its potential application for challenging endodontic diagnostic tasks should be further explored^15,16^. Interestingly, the performance of PCCT was superior to SR- 3D Accuitomo 170 and both SR- and HR- NewTom VGi evo, which further strengthens the fact that it could be regarded as a plausible diagnostic tool. Based on this great performance, PCCT data acquired for other clinical reasons could be used to assess fine endodontic structures, making it unnecessary for the patient to undergo additional CBCT scans.

Amongst different technical parameters of 3D imaging modalities, spatial resolution holds a vital position for the assessment of endodontics diagnostic tasks. It is defined as the ability of an imaging device to represent fine details belonging to the scanned object^17^. One of the main factors influencing this parameter is the voxel size, as tomographic images acquired with a small voxel size have a higher spatial resolution^17,18^. It could be hypothesized that PCCT would perform unsatisfactorily due to a larger voxel size compared to the tested CBCT devices regardless of the acquisition protocol. It should be kept in mind that this parameter is usually mistaken as the only factor influencing the spatial resolution^17^. On the contrary, the effective spatial resolution is a sum of several device-specific technical parameters, such as reconstruction filter technique, focal spot size, number of image basis projections, and magnification factor^2,19^. Hence, the sum of these technical parameters justified the excellent performance of PCCT for detecting challenging endodontic tasks.

To our knowledge, only two studies exist assessing the application of PCCT for visualizing the dentoalveolar region^9,20^. Vanden Broeke et al. evaluated the feasibility of using PCCT to detect accessory root canals and the expression of metal artefacts generated by dental implants compared to CBCT and micro-CT imaging^20^. Although the authors obtained promising results, their findings should be interpreted with caution as the qualitative analysis was performed using extracted teeth. Unlike present study, where a phantom covered with soft-tissue equivalent was used which could be considered more acceptable for replicating a clinical scenario. In another study, the ability of PCCT was investigated for qualitatively delineating cortical and medullary bone, root canals, periodontal space, and apical osteolysis using cadaveric heads^9^. In addition, the authors also assessed the device’s performance for measuring vertical bone loss and buccal cortical plate thickness. Based on their findings, PCCT showed an excellent performance which was comparable to that of a CBCT device.

Overall, our findings revealed an excellent performance with PCCT and HR 3D Accuitomo 170. In addition, HR- 3D Accuitomo 170 also showed better performance compared to HR- NewTom VGi evo. This contrast in performance could be justified by the inherent differences in the technical characteristics of each CBCT device, where HR 3D Accuitomo 170 has a smaller voxel size and FOV and higher signal-to-noise ratio than NewTom VGi evo. Our findings were also consistent with a prior study, where the authors compared the performance of 10 CBCT device and found HR- 3D Accuitomo 170 to be the most optimal for the endodontic assessment of fine anatomic structures^2^.

Currently, CBCT is considered as a radiological standard for assessing tooth-related fine anatomical structures^1,2,19^. Hence, the present study was conducted in an essence to observe whether PCCT would act as a reliable alternative to state-of-the-art CBCT devices. The findings suggest that PCCT could be placed at the same level as a HR CBCT device due to the similarities in performance for assessing most of the endodontic conditions. At the same instance, it is noteworthy that effective radiation dose of PCCT is higher compared to both SR- and HR- CBCT protocols^21^. Hence, PCCT manufacturers are encouraged to develop optimized low dose acquisition protocols and to observe the impact of decreased dose on dental diagnostic tasks. Based on the acquisition protocols adopted in the current study, PCCT showed an estimated adult effective dose ranging from 6.5 to 85-fold higher compared to the tested CBCT protocols. It is crucial to highlight that the intention of the present study was not to indicate PCCT as a replacement for CBCT devices since HR- 3D Accuitomo 170 showed similar performance at a significantly lower effective radiation dose. In fact, the main goal was to address the robustness of PCCT imaging for appropriate evaluation of challenging endodontic diagnostic tasks. As this study confirmed the outstanding performance of PCCT for appropriate or acceptable visualization of fine endodontic structures and root cracks, it could act as a reliable imaging source for endodontic tasks in patients who have previously underwent PCCT imaging of the head and neck region indicated for other medical reasons. Hence, inhibiting the need for CBCT scanning and in turn further reducing the patient's exposure to ionizing radiation^9^.

The present investigation adopted the PCCT standard acquisition protocol for a head scanning, as recommended by the equipment manufacturers. Differently, a previous study^9^ aimed to test the influence of different PCCT acquisition protocols on the visualization capability of imaging tooth structures and apical osteolysis. Interestingly, the high and medium-dose PCCT acquisition protocols showed better visualization capability for cortical bone, spongious bone, and root canals than the low-dose protocol. Considering this statistical difference among the PCCT acquisition protocols, future studies are encouraged to investigate the influence of PCCT scan optimization (i.e. reducing the radiation dose levels) on the visualization capability of challenging endodontic tasks.

A subjective image assessment methodology was opted with standardized and registered CBCT reconstructions in the current study^2^. This allowed to overcome the observer variability and bias associated with dynamic analysis of CBCT images. Even though static image analysis does not represent a clinical norm where images are assessed by dynamic scrolling, it is the most plausible part of methodologies where subjective image quality assessment is the point of interest^11,22–24^.

The main strengths of the study were the application of an anthropomorphic phantom covered with Mix-D for simulating x-ray attenuation as observed in clinical scans and assessment of natural teeth with preexisting challenging endodontic tasks instead of relying on artificially simulated situations^11^. Furthermore, the registration of tested images onto a reference image guaranteed the evaluation at the same anatomical level ^2,25^. At the same instance, certain inherent limitations also existed, such as absence of motion artefacts. In a clinical scenario, motion artefacts are commonly observed which are responsible for decreasing the image quality^26,27^. However, it could be hypothesized that this effect would be more pronounced with CBCT imaging since it is usually performed with the patient standing in an upright position, unlike PCCT imaging which is acquired with the patient in prone position. Future comparative investigations are recommended to investigate the impact of motion artefacts on images acquired with both CBCT and PCCT devices.

## Conclusions

In conclusion, PCCT and HR 3D Accuitomo 170 showed similar performance compared to an industrial micro-CT scanner for appropriate visualization of fine endodontic structures when grouped together. Moreover, HR CBCT protocol was found to be superior for root crack visualization compared to both PCCT and SR and HR NewTom VGi evo protocols.

## Data Availability

The datasets used and/or analysed during the current study available from the corresponding author on reasonable request.
